# Epidemiological, Clinical, and Therapeutic Features of Colon Cancer in the General Surgery Department of the National Institute of Hepato-Virology (INHV) in Nouakchott, Mauritania: A Series of 40 Cases

**DOI:** 10.7759/cureus.102916

**Published:** 2026-02-03

**Authors:** Ahmed Bezeid Hamine, Abdellahi Abed, Mohamed lemine Sow, Ahmed El Heiba Mamina, Ahmedou Moulaye Idriss

**Affiliations:** 1 Departement of Surgery, Faculty of Medicine, Pharmacy, and Odontostomatology, University of Nouakchott, Nouakchott, MRT; 2 Department of Radiology, Faculty of Medicine, Pharmacy, and Odontostomatology, University of Nouakchott, Nouakchott, MRT; 3 Department of Radiology, Hospital Center of Speciality, Nouakchott, MRT; 4 Department of Medical Oncology, University of Nouakchott, Nouakchott, MRT; 5 Faculty of Medicine, University of Nouakchott, Nouakchott, MRT; 6 Anatomy Laboratory, Faculty of Medicine, Pharmacy, and Odontostomatology, University of Nouakchott, Nouakchott, MRT

**Keywords:** clinical presentation, colon cancer, colorectal surgery, epidemiology, mauritania, national institute of hepato-virology, treatment

## Abstract

Background and objectives

In low- and middle-income countries, colon cancer is a growing public health concern and is often diagnosed at an advanced stage. In Mauritania, local data remain scarce. This retrospective, hospital-based descriptive study aimed to describe the epidemiological, clinical, and therapeutic characteristics of surgically managed colon cancer cases in the general surgery department of the National Institute of Hepato-Virology (INHV), Nouakchott, Mauritania, a public tertiary referral hospital serving the general population and receiving referrals from Nouakchott and other regions of the country.

Methods

We conducted a retrospective, descriptive study including 40 patients who underwent surgery for colon cancer between 2020 and 2023. Variables collected included age, sex, medical and surgical history, presenting symptoms, diagnostic delay, tumor location, and therapeutic modalities. Data were extracted from medical records and analyzed using SPSS version 27 (IBM Corp., Armonk, NY).

Results

The mean age was 56.3 ± 14.2 years (range: 30-88). Males accounted for 65% (26 men, 14 women), with a sex ratio of 1.86. Colon cancer accounted for 4% of all admissions in the General Surgery Department at INHV during the study period (department-level denominator). Abdominal pain was the most frequent symptom (19 patients, 47.5%), followed by bowel obstruction in six patients (15.0%) and lower gastrointestinal bleeding in five patients (12.5%). The mean delay between symptom onset and diagnosis was 2.12 months (range: 1-12.5). Tumors were more frequently located in the right colon (22 cases, 55.0%) than in the left colon (18 cases, 45.0%). Laparotomy was performed in 36 cases (90.0%) and laparoscopy in four cases (10.0%). Right hemicolectomy was the most common procedure (15 patients, 37.5%), followed by left hemicolectomy (11 patients, 27.5%). Segmental colectomy was performed in five patients (12.5%), and palliative-intent surgery in nine patients (22.5%). Adjuvant chemotherapy was administered to nine patients (22.5%), mainly for advanced tumors.

Conclusion

In this Mauritanian hospital-based series, colon cancer predominantly affected middle-aged adults with a male predominance. Abdominal pain and bowel obstruction were the most common presenting symptoms. Most patients underwent curative-intent surgery; however, access to early diagnosis and adjuvant oncologic treatment remains limited. Given the retrospective descriptive design and limited standardized follow-up, survival and recurrence outcomes were not formally analyzed. These findings highlight the need to promote early detection, improve access to colonoscopy, and strengthen multidisciplinary management.

## Introduction

Colon cancer is a malignant neoplasm arising from the epithelial lining of the colon, extending from the ileocecal valve to the rectosigmoid junction. It develops through a multistep process involving progressive genetic and morphological alterations that lead to uncontrolled proliferation and malignant transformation. Colon cancer is a major public health concern worldwide because of its high incidence and mortality [1,2].

Globally, it ranks among the most frequently diagnosed malignancies and predominantly affects individuals older than 50 years [3]. Established risk factors include advanced age, overweight/obesity, physical inactivity, tobacco use, unhealthy lifestyle habits, and familial or genetic predisposition.

Despite progress in diagnostic techniques, colon cancer is often diagnosed at an advanced stage, particularly in low- and middle-income countries where limited access to healthcare and diagnostic tools may delay evaluation. Surgical resection remains the cornerstone of curative treatment, and minimally invasive approaches such as laparoscopy can reduce postoperative morbidity in selected patients [4]. Adjuvant chemotherapy improves outcomes in patients with stage III disease.

Early detection through screening (immunochemical fecal occult blood testing followed by colonoscopy) improves prognosis by enabling diagnosis at earlier stages [5]. However, local epidemiological and management data from Mauritania remain limited. Therefore, this study aimed to describe the epidemiological characteristics, clinical presentation, and therapeutic management of colon cancer patients treated in Mauritania and to compare our findings with the existing literature.

## Materials and methods

Study design

This was a retrospective, descriptive study including 40 patients who underwent surgical treatment for colon cancer over a four-year period, from January 2020 to December 2023.

Study setting

The study was conducted in the General Surgery Department of the National Institute of Hepatology and Virology, a tertiary referral center in Nouakchott, Mauritania.

Study population

All patients admitted to the General Surgery Department and surgically managed for colon cancer during the study period were retrospectively reviewed.

Inclusion and exclusion criteria

Patients were included if they had a tumor located in the colon, extending from the cecum to the rectosigmoid junction, with histopathological confirmation of colon cancer on the surgical specimen, and if they underwent surgical management during the defined study period.

Patients were excluded if there was no histological confirmation of malignancy on the surgical specimen, if the colonic lesion represented metastatic disease from another primary tumor, or if the medical records were incomplete or unusable for analysis.

Patient selection process

**Figure 1:**
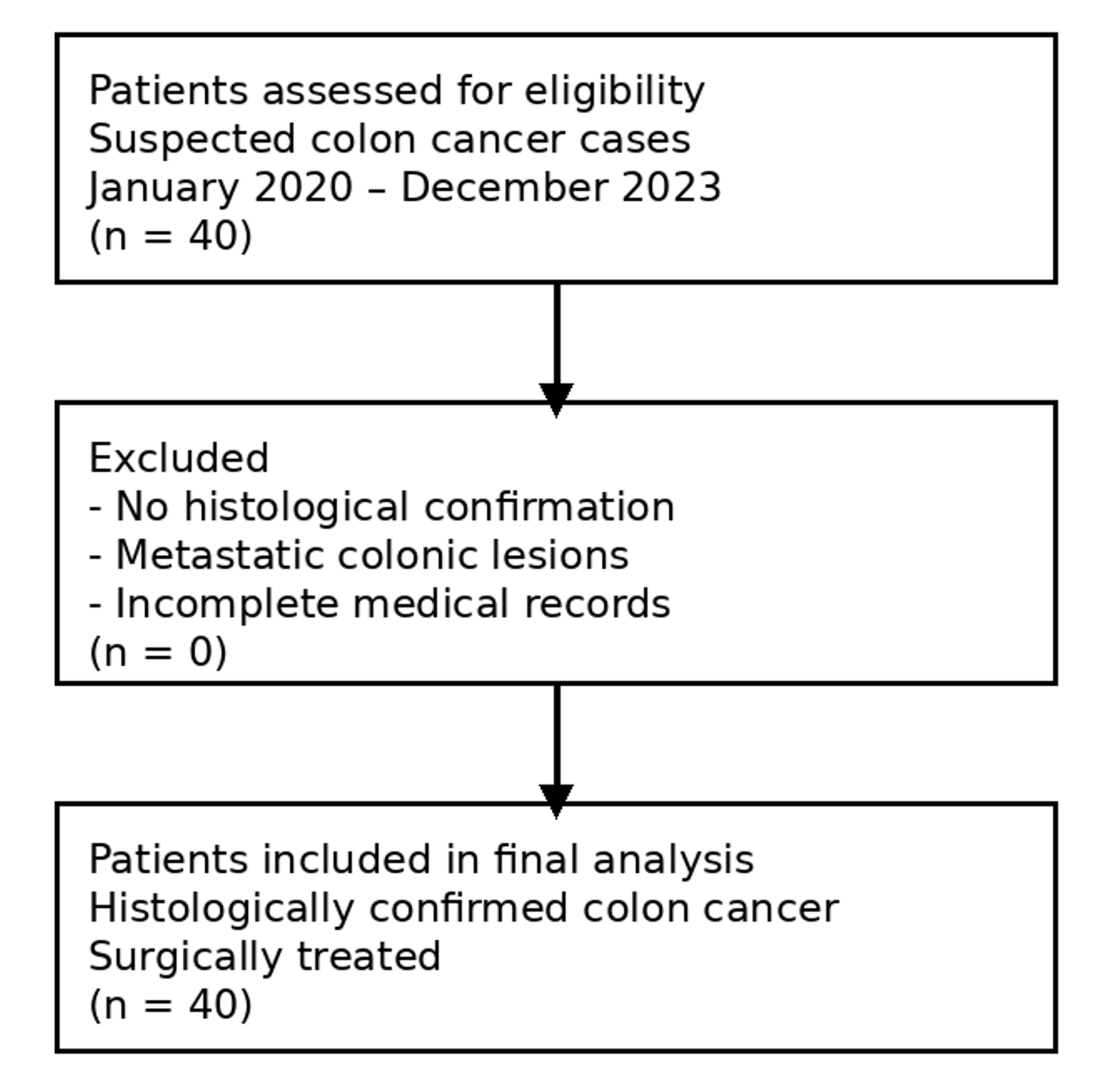
Flowchart of patient selection.

The process of patient selection, including inclusion and exclusion criteria, is summarized in Figure 1.

Data collection

Data were collected retrospectively from patients’ medical records using a standardized data collection form. The collected variables included epidemiological characteristics, clinical presentation, endoscopic findings (when available), histopathological results, radiological staging, and therapeutic management.

Therapeutic management (decision criteria)

Surgical procedures were selected according to tumor location, intraoperative findings, and resectability, following standard oncologic principles. Palliative-intent surgery was performed when curative resection was not feasible, most commonly to relieve bowel obstruction and/or control bleeding. Adjuvant chemotherapy was prescribed mainly for advanced tumors according to the treating oncologist's assessment and local availability of oncologic care.

Missing data

Due to the retrospective design, some variables were not available in all records. Missing or undocumented variables were recorded as missing and excluded from denominator calculations for the corresponding percentages. No imputation was performed.

Statistical analysis

Statistical analysis was performed using SPSS version 27 (IBM Corp., Armonk, NY). Descriptive statistics were used to summarize the data. Categorical variables were expressed as frequencies and percentages, while continuous variables were expressed as means and ranges. Text processing and figure preparation were carried out using Microsoft Word 2019 (Microsoft Corporation, Redmond, WA).

Ethical considerations

The study protocol was reviewed and approved by the institutional ethics committee of the National Institute of Hepato-Virology (INHV), Nouakchott, Mauritania. As this was a retrospective chart review using anonymized data, the requirement for written informed consent was waived. All data were handled confidentially and analyzed in a de-identified manner.

## Results

Epidemiological data

During the study period from 2020 to 2023, colon cancer accounted for approximately 4% of all hospitalizations in the General Surgery Department of the National Institute of Hepato-Virology, with an average of 10 cases per year (N = 40).

The mean age of the patients was 56.3 years (range: 30-88 years). There was a clear male predominance, with 26 men (65%) and 14 women (35%), resulting in a sex ratio of 1.86.

**Figure 2:**
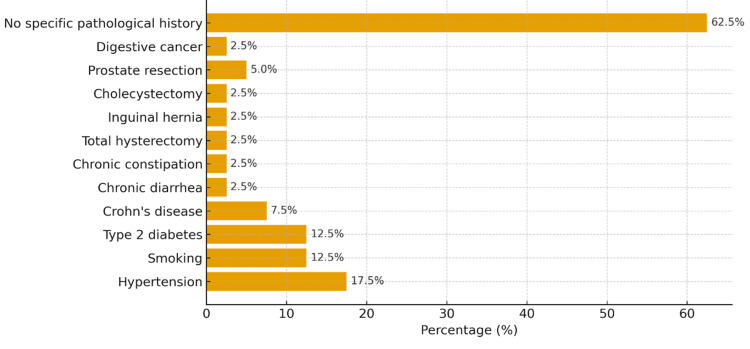
Personal and family medical histories of the patients.

Personal and family medical histories were recorded for all patients (N = 40) and are summarized in Figure 2. A subset of patients reported a family history of colon neoplasia or other gastrointestinal malignancies, suggesting a possible role of genetic predisposition.

Clinical aspects

Presenting Symptoms of Colonic Cancer

Abdominal pain was the most common presenting symptom, reported in 19 patients (47.5%), and was either localized or diffuse (Table 1). Bowel obstruction, manifested by cessation of stool and gas, was observed in six patients (15%), while vomiting occurred in three patients (7.5%). Lower gastrointestinal bleeding (rectorrhagia) was noted in five patients (12.5%). Other symptoms included anemia in four patients (10%), dehydration in two patients (5%), and fever in one patient (2.5%).

**Table 1:** Distribution of patients according to presenting symptoms of colonic cancer.

Symptom	Number	Percentage (%)
Abdominal pain	19	47.5
Cessation of stool and gas	6	15.0
Lower gastrointestinal bleeding	5	12.5
Vomiting	3	7.5
Dehydration	2	5.0
Anemia	4	10.0
Fever	1	2.5
Total	40	100.0

Diagnostic Delay

The mean delay between symptom onset and diagnosis was 2.12 months (range: one month to 12 months and 17 days) (Table 2).

**Table 2:** Distribution of patients according to the delay between the first symptom and diagnosis.

Diagnostic delay	Number	Percentage (%)
0–1 month	22	55.0
2–3 months	7	17.5
4–5 months	3	7.5
6–7 months	3	7.5
8–9 months	3	7.5
10–11 months	1	2.5
>12 months	1	2.5
Total	40	100.0

​​​​​​*Physical Examination*

An altered general condition, characterized by weight loss, asthenia, and anorexia, was observed in 35 patients (87.5%).

Endoscopic findings

Lower gastrointestinal endoscopy was performed in 34 patients (85%). Among these patients, endoscopic findings revealed stenosing lesions in 15 cases (44.1%), ulcerative-proliferative lesions in 13 cases (38.2%), purely ulcerative lesions in three cases (8.8%), and proliferative (mass-forming) lesions in two cases (5.9%). In the remaining six patients (15%), colonoscopy was not performed or was not documented in the medical records, which may reflect emergency presentation (e.g., acute bowel obstruction requiring urgent surgical management) and/or limited availability or scheduling constraints in routine practice. In these cases, the diagnosis was established based on the overall clinical evaluation and cross-sectional imaging when available, and was definitively confirmed by histopathology.

Histological types

Histological examination showed that well-differentiated Lieberkühnian adenocarcinoma was the predominant subtype, accounting for 32 cases (80%). Poorly differentiated adenocarcinoma was identified in four patients (10%), mucinous adenocarcinoma in three patients (7.5%), and one patient (2.5%) was diagnosed with colonic lymphoma.

Evaluation of tumor extension

All patients underwent contrast-enhanced computed tomography (CT) for tumor staging. CT imaging demonstrated a localized and resectable tumor in 31 patients (77.5%), while nine patients (22.5%) presented with locally advanced disease. Tumors were located in the right colon in 22 patients (55%) and in the left colon in 18 patients (45%).

Treatment

All 40 patients underwent surgical management (Table 3). Curative-intent surgery was performed in 31 patients (77.5%), including right hemicolectomy in 15 patients (37.5%), left hemicolectomy in 11 patients (27.5%), and segmental colonic resection in five patients (12.5%). Palliative surgery was performed in nine patients (22.5%), all of whom underwent diverting colostomy. Laparoscopic surgery was used in four patients (10%), while 36 patients (90%) underwent open surgery. Adjuvant chemotherapy was indicated in nine patients with high-risk pathological or clinical features.

**Table 3:** Distribution of patients according to surgical procedures.

Surgical procedure	Number	Percentage (%)
Right hemicolectomy	15	37.5
Left hemicolectomy	11	27.5
Segmental resection	5	12.5
Colostomy	9	22.5
Total	40	100

## Discussion

Epidemiological aspects

Frequency

Colorectal cancer represents a major public health challenge in high-income countries, where it ranks among the most common malignant tumors and remains a leading cause of cancer-related mortality worldwide [1,2,6]. In contrast, lower incidence rates have traditionally been reported in Africa, as well as in South America and parts of Asia [7-9].

Data from hospital-based and regional registries in Africa nevertheless suggest a progressive increase in the burden of colorectal cancer, reflecting epidemiological transition, lifestyle changes, and improved diagnostic capabilities [8]. These observations indicate that colorectal cancer is emerging as a significant health concern in our setting.

Age

The mean age at diagnosis in our cohort was 56.3 years, which is younger than that reported in Western countries such as France, where colorectal cancer is typically diagnosed in the seventh decade of life [10,11].

Several African series, particularly from Senegal, have reported even younger ages at diagnosis, often around the fifth decade [8,12]. Our findings, therefore, appear intermediate between Western and Sub-Saharan African profiles and are comparable to data reported from Morocco [4,13,14]. This age distribution may reflect demographic characteristics, genetic factors, and evolving lifestyle patterns in North and West Africa.

Sex

A male predominance in colorectal cancer has been widely reported in European and African studies. Moroccan series have similarly described a sex ratio close to unity, consistent with observations from Senegal and other African countries [8,12].

In our study, male predominance was more marked, with a sex ratio of 1.86. This finding aligns with most African series and may be explained by differences in exposure to environmental risk factors as well as variations in healthcare-seeking behavior between men and women.

Etiological Factors

Colorectal cancer may arise through sporadic or hereditary pathways [15-17]. Several inherited syndromes are associated with a markedly increased risk, including familial adenomatous polyposis, Lynch syndrome, Peutz-Jeghers syndrome, juvenile polyposis, Cowden syndrome, Gardner syndrome, and Turcot syndrome [18-22].

Familial adenomatous polyposis is characterized by germline mutations in the APC gene and confers an almost inevitable risk of colorectal cancer if left untreated [23-25]. Lynch syndrome accounts for a minority of colorectal cancers and is associated with early onset, proximal tumor location, mucinous histology, and microsatellite instability related to mismatch repair deficiency [26-28]. These inherited conditions highlight the importance of targeted screening and surveillance strategies in high-risk populations [29].

Clinical aspects

Diagnostic Delay

Colorectal cancer frequently remains asymptomatic for prolonged periods and is often diagnosed at an advanced stage, particularly when complications occur. In high-income countries, diagnostic delays are generally shorter due to organized screening and better access to healthcare services [11,30].

In African settings, longer diagnostic delays have been reported. Studies from the Central African Republic have shown that a substantial proportion of patients present several months or years after symptom onset [31]. Western series have also documented variable diagnostic delays, ranging from a few weeks to several months [32].

In our study, the mean diagnostic delay was 2.12 months, which appears shorter than that reported in many African series. This may reflect earlier symptom recognition or diagnosis prompted by acute complications.

Presenting Symptoms

In our cohort, abdominal pain, bowel obstruction, lower gastrointestinal bleeding, anemia, vomiting, and general symptoms were the most common presenting features.

Previous African studies have similarly reported abdominal pain, intestinal obstruction, and rectal bleeding as frequent modes of presentation, often reflecting advanced disease at diagnosis [30]. Differences in symptom frequencies across studies may be related to variations in tumor stage and healthcare access.

Main Clinical Signs

Abdominal pain is a common symptom of colorectal cancer and may present with variable intensity and localization. It is frequently associated with partial or complete bowel obstruction and has been reported in a substantial proportion of patients across series [3].

In our study, abdominal pain was the predominant clinical sign. Obstructive symptoms occurred in a proportion of patients comparable to that reported in other North African series [33]. Rectal bleeding was less frequent in our cohort than in some published studies [33,34].

Endoscopic Findings

Lower gastrointestinal endoscopy plays a central role in the diagnosis of colorectal cancer by allowing direct visualization of lesions, biopsy sampling, and assessment of tumor morphology. In our series, endoscopy was performed in 34 patients, representing 85% of the study population, a rate comparable to that reported in other African studies, where incomplete examinations are often related to obstructive complications or poor patient tolerance.

Among patients who underwent endoscopy (n = 34), stenosing lesions were observed in 15 cases (44.1%), while ulcerative-proliferative lesions were identified in 13 cases (38.2%). Purely ulcerative lesions were found in three patients (8.8%), and proliferative or mass-forming lesions in two patients (5.8%). These findings reflect advanced disease at diagnosis and are consistent with patterns described in low- and middle-income countries [3,30,31,33].

Histological Types

Adenocarcinoma remains the predominant histological type of colorectal cancer worldwide. In our study, well-differentiated adenocarcinoma was the most frequent subtype, consistent with findings from African and Western series [3,13,14].

Poorly differentiated and mucinous adenocarcinomas were less common but are known to be associated with more aggressive behavior and poorer prognosis. Mucinous histology has been reported more frequently in younger patients and in hereditary colorectal cancer syndromes, particularly Lynch syndrome [20,22]. Primary colonic lymphoma remains rare and has been sporadically reported in the literature [3].

Evaluation of Tumor Extension

Contrast-enhanced computed tomography is essential for preoperative staging, allowing assessment of local extension, nodal involvement, and distant metastases. Its systematic use in our cohort reflects adherence to international recommendations.

The proportion of localized and potentially resectable tumors in our series appears higher than that reported in some African studies, where advanced disease at diagnosis is common [8,31]. Right-sided tumors slightly predominated, a distribution that has been increasingly reported in the literature and may be associated with genetic and age-related factors [20-22].

Therapeutic aspects

Surgical Strategy and Techniques

For elective surgery of colorectal cancer, current evidence supports preoperative mechanical bowel preparation combined with oral antibiotics. Meanwhile, for patients with varied degrees of intestinal stenosis, an individualized protocol is required to avoid adverse events [35].

Research shows that administration of prophylactic antibiotics before colorectal surgery prevents postoperative surgical wound infection. The best antibiotic choice, timing of administration, and route of administration remain undetermined [36].

The principles of oncologic colectomy include en bloc tumor resection with adequate margins and lymphadenectomy based on vascular anatomy [37]. Early ligation of vascular pedicles has been advocated to minimize tumor dissemination [38].

Although laparotomy remains the most commonly used approach in low-resource settings, laparoscopic colectomy has demonstrated comparable oncologic outcomes with improved short-term recovery in selected patients [34,37].

Types of Colectomy

Right hemicolectomy is commonly performed for right-sided colon tumors [39]. In our study, this procedure was the most frequently performed, with a proportion comparable to that reported in other series [40,41].

Left hemicolectomy and segmental colectomy frequencies vary widely across studies depending on tumor location and stage [8,42,43]. Our findings fall within the ranges reported in the literature.

Palliative Surgery

Palliative surgery is indicated for unresectable tumors, most often to relieve obstruction or bleeding [44]. Reported rates vary across series [42]. In our study, the proportion of patients undergoing palliative procedures was comparable to that reported in similar settings.

Chemotherapy

Chemotherapy plays an important role in adjuvant and palliative settings [45,46]. In high-income countries, a substantial proportion of patients receive systemic treatment [47]. In our cohort, chemotherapy use was lower, likely reflecting limited access to oncology services and advanced disease at presentation.

Radiotherapy and Immunotherapy

Radiotherapy has no established role in colon cancer management, unlike rectal cancer. Immunotherapy targeting microsatellite instability-high (MSI-H) or deficient mismatch repair (dMMR) tumors represents an emerging therapeutic option and may significantly influence future treatment strategies.

Limitations

This study has limitations inherent to its retrospective, single-center design. Postoperative morbidity and mortality outcomes were not systematically recorded in the available medical records and could not be reported using a standardized classification (e.g., Clavien-Dindo). In addition, some clinical variables may have been under-reported due to incomplete documentation. The relatively small sample size (n = 40) may also limit the precision and generalizability of our findings. Future prospective studies with standardized perioperative outcome reporting and follow-up are warranted to better quantify postoperative complications and outcomes in our setting. Larger multicenter studies are needed to provide more representative national estimates and to better characterize staging, treatment patterns, and outcomes of colon cancer in Mauritania.

## Conclusions

Colon cancer continues to pose a substantial and increasing health challenge in low- and middle-income nations, where diagnosis frequently experiences delays. This Mauritanian series indicated that the disease primarily impacted middle-aged people, with a male predominance, and most patients exhibited advanced clinical manifestations, including abdominal discomfort and intestinal blockage. Surgical intervention continued to be the fundamental approach of treatment, with curative resection attainable in a significant number of instances. Nonetheless, restricted access to early diagnostic instruments, especially colonoscopy, and to supplementary oncological treatments persists in affecting overall management.

These findings highlight the pressing necessity to enhance colorectal cancer screening protocols, augment access to endoscopic and imaging services, and foster multidisciplinary collaboration to optimize patient outcomes. Additional prospective, multicenter studies are necessary to more accurately delineate the epidemiological profile and enhance standardized care protocols for colon cancer in Mauritania.

## References

[1] Ngasseu P, Dieye M, Veronique-Baudin J (2007). Colorectal cancers in Martinique: incidence and mortality rates over a period of 20 years. (Article in French). Rev Epidemiol Sante Publique.

[2] Belhamidi MS, Sinaa M, Kaoukabi A, Krimou H, Menfaa M, Sakit F, Choho A (2018). Epidemiological and pathological profile of colorectal cancer: about 36 cases. (Article in French). Pan Afr Med J.

[3] Rawla P, Sunkara T, Barsouk A (2019). Epidemiology of colorectal cancer: incidence, mortality, survival, and risk factors. Prz Gastroenterol.

[4] De Brauer C, Bousquet PJ, Lafay L (2024). Cancers: incidence and survival in metropolitan France. (Article in French). Rev Prat.

[5] Joachim C, Macni J, Drame M, Pomier A, Escarmant P, Veronique-Baudin J, Vinh-Hung V (2019). Overall survival of colorectal cancer by stage at diagnosis: data from the Martinique Cancer Registry. Medicine (Baltimore).

[6] Henrikson NB, Webber EM, Goddard KA (2015). Family history and the natural history of colorectal cancer: systematic review. Genet Med.

[7] Graham A, Adeloye D, Grant L, Theodoratou E, Campbell H (2012). Estimating the incidence of colorectal cancer in Sub-Saharan Africa: a systematic analysis. J Glob Health.

[8] Imad FE, Drissi H, Tawfiq N, Bendahhou K, Jouti NT, Benider A, Radallah D (2019). Epidemiological, nutritional and anatomopathological features of patients with colorectal cancer in the greater Casablanca region. Pan Afr Med J.

[9] Mármol I, Sánchez-de-Diego C, Pradilla Dieste A, Cerrada E, Rodriguez Yoldi MJ (2017). Colorectal carcinoma: a general overview and future perspectives in colorectal cancer. Int J Mol Sci.

[10] Santucci C, Carioli G, Bertuccio P (2020). Progress in cancer mortality, incidence, and survival: a global overview. Eur J Cancer Prev.

[11] Sassi A, Bacha D, Talbi G (2017). Colon adenocarcinoma and synchronous type 1 papillary renal cell carcinoma: a unique association. (Article in French). Pan Afr Med J.

[12] Marley AR, Nan H (2016). Epidemiology of colorectal cancer. Int J Mol Epidemiol Genet.

[13] Imad FE, Drissi H, Tawfiq N, Bendahhou K, Benider A, Radallah D (2020). A case-control study on dietary risk factors for colorectal cancer in Morocco. Pan Afr Med J.

[14] Tuca A, Guell E, Martinez-Losada E, Codorniu N (2012). Malignant bowel obstruction in advanced cancer patients: epidemiology, management, and factors influencing spontaneous resolution. Cancer Manag Res.

[15] Charifa A, Jamil RT, Sathe NC, Zhang X (2025). Gardner syndrome. StatPearls.

[16] Jo WS, Chung DC (2005). Genetics of hereditary colorectal cancer. Semin Oncol.

[17] Magaña M, Landeta-Sa AP, López-Flores Y (2022). Cowden disease: a review. Am J Dermatopathol.

[18] Murphy N, Norat T, Ferrari P (2012). Dietary fibre intake and risks of cancers of the colon and rectum in the European prospective investigation into cancer and nutrition (EPIC). PLoS One.

[19] Ankouane F, Noah DN, Nonga BN, Tagni-Sartre M, Modjo G, Ndam EC (2014). Endoscopic resection of colorectal polyps pedicles using a releasable lasso over chrome catgut thread: an alternative to conventional polypectomy? Report of a case series. (Article in French). Pan Afr Med J.

[20] Paz-Bouza JI, Noriega de Castro J, Abad Hernández MM, Galindo Villardón P, Pérez-Ramos C, Muñoz Torres E (1990). Colorectal cancer: retrospective analysis of 762 cases. (Article in Spanish). An Med Interna.

[21] Hryhorowicz S, Kaczmarek-Ryś M, Lis-Tanaś E (2022). Strong hereditary predispositions to colorectal cancer. Genes.

[22] Peltomäki P, Nyström M, Mecklin JP, Seppälä TT (2023). Lynch syndrome genetics and clinical implications. Gastroenterology.

[23] Zorluoglu A, Yilmazlar T, Ozguc H, Bagcivan E, Guner O (2004). Colorectal cancers under 45 years of age. Hepatogastroenterology.

[24] Arumugam PJ, Joseph A, Sweerts M, Haray PN (2002). Severe dysplastic lesions in the colon - How aggressive should we be?. Colorectal Dis.

[25] Zhu QC, Shen RR, Qin HL, Wang Y (2014). Solitary rectal ulcer syndrome: clinical features, pathophysiology, diagnosis and treatment strategies. World J Gastroenterol.

[26] Ouryemchi M, Jabi R, Soussan H, Najioui Y, Bouziane M (2021). Degenerative ulcerative colitis after one year of evolution in a 20-year-old patient. Cureus.

[27] Botteri E, Iodice S, Bagnardi V, Raimondi S, Lowenfels AB, Maisonneuve P (2008). Smoking and colorectal cancer: a meta-analysis. JAMA.

[28] Song M, Garrett WS, Chan AT (2015). Nutrients, foods, and colorectal cancer prevention. Gastroenterology.

[29] Ye P, Xi Y, Huang Z, Xu P (2020). Linking obesity with colorectal cancer: epidemiology and mechanistic insights. Cancers.

[30] Katsaounou K, Nicolaou E, Vogazianos P (2022). Colon cancer: from epidemiology to prevention. Metabolites.

[31] Youssouf O, Odjo J, George S, Siolo BE, Koffi B (2023). Epidemiological and clinical aspects of colorectal cancers in Bangui. (Article in French). ESJ.

[32] Yoon JW, Lee SH, Ahn BK, Baek SU (2008). Clinical characteristics of multiple primary colorectal cancers. Cancer Res Treat.

[33] Bray F, Parkin DM (2022). Cancer in sub-Saharan Africa in 2020: a review of current estimates of the national burden, data gaps, and future needs. Lancet Oncol.

[34] Weissman S, Sebrow J, Gonzalez HH (2019). Diagnosis of primary colorectal carcinoma with primary breast cancer: associations or connections?. Cureus.

[35] Dou RX, Zhou ZL, Wang JP (2022). Bowel preparation before elective surgery for colorectal cancer. (Article in Chinese). Zhonghua Wei Chang Wai Ke Za Zhi.

[36] Nelson RL, Gladman E, Barbateskovic M (2014). Antimicrobial prophylaxis for colorectal surgery. Cochrane Database Syst Rev.

[37] Gu J (2022). Resection margin of colorectal cancer surgery. (Article in Chinese). Zhonghua Wei Chang Wai Ke Za Zhi.

[38] Millan M, Merino S, Caro A, Feliu F, Escuder J, Francesch T (2015). Treatment of colorectal cancer in the elderly. World J Gastrointest Oncol.

[39] Crippa J, Grass F, Achilli P (2021). Surgical approach to transverse colon cancer: analysis of current practice and oncological outcomes using the National Cancer Database. Dis Colon Rectum.

[40] Tuech JJ, Gangloff A, Di Fiore F (2020). Strategy for the practice of digestive and oncological surgery during the COVID-19 epidemic. J Visc Surg.

[41] Abdelrazeq AS, Scott N, Thorn C, Verbeke CS, Ambrose NS, Botterill ID, Jayne DG (2008). The impact of spontaneous tumour perforation on outcome following colon cancer surgery. Colorectal Dis.

[42] Helfritzsch H, Böhm B, Thiele M, Altendorf-Hoffmann A, Scheele J (2002). Results of the surgical therapy in advanced colorectal cancer. (Article in German). Zentralbl Chir.

[43] Iancu A, Hardy PY, Coimbra C, Joris J (2021). Ambulatory laparoscopic colectomy: first experiences at the CHU of Liège. Rev Med Liege.

[44] Darr H, Abbas MA (2020). Stenting as a bridge to surgery or a palliative treatment. Clin Colon Rectal Surg.

[45] Saltz LB, Minsky B (2002). Adjuvant therapy of cancers of the colon and rectum. Surg Clin North Am.

[46] Wolpin BM, Meyerhardt JA, Mamon HJ, Mayer RJ (2007). Adjuvant treatment of colorectal cancer. CA Cancer J Clin.

[47] Lopez-Trabada D, Philippe A, Sorbere M, Lusardi V, Boussion H (2022). Medical oncological treatment of colorectal cancer in the elderly. (Article in French). Soins Gerontol.

